# Identifying heavy health care users among primary care patients with chronic non-cancer pain

**DOI:** 10.1080/24740527.2017.1326088

**Published:** 2017-07-06

**Authors:** Elie Antaky, Lyne Lalonde, Mireille E. Schnitzer, Élisabeth Martin, Djamal Berbiche, Sylvie Perreault, David Lussier, Manon Choinière

**Affiliations:** a Faculty of Pharmacy, Université de Montréal, Montreal, Quebec, Canada; b Centre de recherche du Centre hospitalier de l’Université de Montréal (CRCHUM), Montreal, Quebec, Canada; c Sanofi Aventis Endowment Chair in Ambulatory Pharmaceutical Care, Université de Montréal, Montreal, Quebec, Canada; d Sanofi Aventis Endowment Research Chair in Optimal Drug Use, Université de Montréal, Montreal, Quebec, Canada; e Institut universitaire de gériatrie de Montréal, Montreal, Quebec, Canada; f Division of Geriatric Medicine and Alan Edwards Centre for Research on Pain, McGill University, Montreal, Quebec, Canada; g Department of Anesthesiology, Faculty of Medicine, Université de Montréal, Montreal, Quebec, Canada

**Keywords:** chronic non-cancer pain, primary care, predictors of heavy health care use, Akaike information criterion

## Abstract

**Objective**: The objective of this study was to identify biopsychosocial factors predicting primary care chronic non-cancer pain (CNCP) patients’ risk of being heavy health care users.

**Methods**: Patients reporting moderate to severe CNCP for at least 6 months with an active analgesic prescription from a primary care physician were recruited in community pharmacies. Recruited patients completed questionnaires documenting biopsychosocial characteristics. Using administrative databases, direct costs were estimated for health care services used by each patient in the year preceding and following the recruitment. Heavy health care users were defined as patients in the highest annual direct health care costs quartile. Logistic multivariate regression models using the Akaike information criterion were developed to identify predictors of heavy health care use.

**Results**: The median annual direct health care cost incurred by heavy health care users (*n* = 63) was CAD (Canadian dollars) 7627, versus CAD 1554 for standard health care users (*n* = 188). The final predictive model of the risks of being a heavy health care user included pain located in the lower body (odds ratio [OR] = 3.03; 95% confidence interval [CI], 1.20–7.65), pain-related disability (OR = 1.24; 95% CI, 1.03–1.48), and health care costs incurred in the year prior to recruitment (OR = 17.67; 95% CI, 7.90–39.48). Variables in the model also included sex, comorbidity, patients’ depression level, and attitudes toward medical pain cure.

**Conclusion**: Patients suffering from CNCP in the lower body and showing greater disability were more likely to be heavy health care users, even after adjusting for previous-year direct health care costs. Improving pain management for these patients could have positive impacts on health care use and costs.

## RÉSUMÉ

**Objectif**: Déterminer les facteurs biopsychosociaux prédictifs du risque d’être un grand utilisateur de services de santé chez les patients souffrant de douleur chronique non cancéreuse (DCNC) suivis en soins de première ligne.

**Méthodologie**: Des patients souffrant d’une DCNC modérée à sévère depuis au moins six mois et bénéficiant d’une ordonnance d’analgésique valide donnée par un médecin de famille ont été recrutés dans des pharmacies communautaires. Les patients recrutés ont ensuite complété des questionnaires documentant leurs caractéristiques biopsychosociales. À l’aide de bases de données administratives, les coûts directs des services de santé utilisés par chaque patient au cours de l’année précédant et suivant son recrutement ont été estimés. Les grands utilisateurs de soins de santé ont été définis comme les patients faisant partie du quartile le plus élevé de coûts directs annuels en soins de santé. Des modèles de régression logistique multivariée utilisant le critère d’information Akaike ont été élaborés afin de déterminer les facteurs prédictifs de grande utilisation des soins de santé.

**Résultats**: Le coût direct annuel médian en soins de santé chez les grands utilisateurs de soins de santé (n = 63) était de 7 627 CAD comparativement à 1 554 CAD pour les utilisateurs de soins de santé réguliers (n = 188). Le modèle prédictif final du risque d’être un grand utilisateur de soins de santé comprenait la douleur au niveau des membres inférieurs (rapport de cotes (RC) = 3,03; intervalle de confiance (IC) à 95 %: 1,20 – 7,65), la réduction de la capacité fonctionnelle liée à la douleur (RC = 1,24; IC à 95 %: 1,03 – 1,48) et les coûts directs en soins de santé au cours de l’année précédant le recrutement (RC = 17,67; IC à 95 %: 7,90 – 39,48). Les variables « sexe », « comorbidité », « dépression » et « attitudes envers la guérison médicale » étaient également retenues dans le modèle.

**Conclusion**: Les patients souffrant de DCNC au niveau des membres inférieurs et présentant une détérioration de la capacité fonctionnelle liée à la douleur étaient plus susceptibles d’être de grands utilisateurs des soins de santé, même après ajustement pour le coût direct en soins de santé pendant l’année précédente. L’amélioration de la prise en charge de la douleur chez ces patients pourrait avoir des répercussions positives sur l’utilisation et le coût des soins de santé.

## Introduction

About one in five people suffers from chronic non-cancer pain (CNCP) in Canada^1–3^ and elsewhere in the world.^4–8^ CNCP is more prevalent in women than in men and is expected to increase dramatically as the population ages.^9^

CNCP is associated with significant costs for patients, their family, and society.^10–13^ In a large Danish study, Jensen et al. found that patients with moderate or severe chronic pain visited their physicians more often (six and nine times/year, respectively) than did those without pain (four times/year).^14^ They were also more likely to be hospitalized (0.8 and 1.6 days/year, versus 0.43 days/year, respectively).^14^ Annual number of pain-related medical visits for CNCP patients were estimated to be eight in Europe^15^ and 17 in the United States,^16^ and average length of hospital stay was 0.3 days in Europe.^15^ In Canada, CNCP patients have been reported to be four times more likely to visit their physician (12.9 versus 3.8 visits) and to stay in hospital six times longer (3.9 versus 0.7 days) than patients without CNCP.^10,17^

Direct health care costs associated with CNCP are substantial.^13^ According to Gaskin and Richard,^13^ the annual cost of CNCP in 2010 in the United States (US$560 to $635 billion) was greater than the annual costs of heart disease (US$309 billion), cancer (US$243 billion), and diabetes (US$188 billion) and nearly 30% higher than the combined cost of cancer and diabetes. Chronic rheumatoid diseases are considered the most expensive group of conditions after cardiovascular diseases^18^ and were ranked among the most expensive chronic pain disorders along with low back pain and osteoarthritis.^19^ A recent study in the province of Quebec (Canada)^11^ calculated the mean annual per patient direct health care cost (2013 values) in a sample of 483 CNCP sufferers to be CAD (Canadian dollars) 9565 (SD = 13 993) based on (1) hospitalizations (CAD 2036; SD = 10 137); (2) emergency room visits (CAD 320; SD = 686); (3) outpatients visits (CAD 604; SD = 541); (4) complementary health care visits, such as physiotherapy, massage therapy, occupational therapy, chiropractic and acupuncture (CAD 4505; SD = 8115); (5) prescribed analgesics and medications to treat drug side effects (CAD 1963; SD = 2978); and (6) over-the-counter analgesics and medications to treat side effects (CAD 136; SD = 601). In a larger and more recent study in another Canadian province (Ontario), the annual incremental cost to manage chronic pain was found to be CAD 1742 per patient (95% confidence interval [CI], $1488–$2020); that is, 51% more than a comparable patient without chronic pain. The largest contributor to this cost was hospitalisation (CAD 514; 95% CI, $364–$683). Costs were higher in patients with more severe pain (CAD 3960; 95% CI, $3186–$4680) and greater activity limitations (CAD 4365; 95% CI, $3631–$5147; 2014 values).^20^

Given the heavy societal and economic burden of CNCP, a thorough understanding of the biopsychosocial factors driving health care costs is of prime interest. Indeed, being able to longitudinally identify CNCP patients most likely to be heavy health care users could be beneficial in order to possibly modify their continuum of care, improve their health outcomes, and thereby reduce their health care costs.

Studies have shown that CNCP patients with depression,^21,22^ anxiety,^23^ sleep problems,^24^ higher pain intensity,^13,25^ more pain-related disability,^11^ and comorbidities^21,26,27^ and those reporting low treatment satisfaction^28,29^ were more likely to use health care resources and generate higher economic costs. However, nearly all of these studies used a cross-sectional design or included only patients with specific CNCP syndromes followed in specialized pain clinics. To our knowledge, no longitudinal studies have been conducted among CNCP patients followed in primary care settings.

The aim of the present longitudinal study was to identify the sociodemographic, psychosocial, and clinical predictors of heavy public health care use among primary care CNCP patients.

## Methods

### Study design

The study design has been described in detail elsewhere.^11^ Briefly, CNCP patients were recruited between May 2009 and January 2010 in community pharmacies (non-hospital based) in various areas in the province of Quebec (Canada). Participants completed a telephone interview and a self-administered questionnaire. Administrative databases of the Quebec Ministère de la santé et des services sociaux (MSSS) (Régie d’Assurance Maladie du Québec (RAMQ), and Maintenance et exploitation des données pour l’étude de la clientèle hospitalière (MED-ÉCHO) were used to document each patient’s health care use in the prerecruitment and postrecruitment year. In Quebec, basic and specialized health care services are covered by the publicly funded RAMQ system; however, this comprehensive coverage does not extend to medications for all patients. Overall, 43% of Quebec’s population aged between 45 and 64 years old and 94% of those ≥65 years have RAMQ coverage for their prescribed medication.^30^

Ethical approval for the study was obtained from the research ethics committees of the Centre intégré de santé et de services sociaux de Laval, the Centre de recherche du Centre hospitalier de l’Université de Montréal, and the “Commission d’accès à l’information” of the Quebec government. Each patient signed an informed consent form and received financial compensation of $25 when both the telephone and self-administered questionnaires were completed. Pharmacists received financial compensation of $50 for every consenting patient.

### Study population

From an atlas published by the MSSS, 499 community pharmacies were identified on the territory of the Réseau universitaire intégré de santé de l’Université de Montréal, which comprises six areas: Mauricie and central Quebec, Laval, Montreal, the Laurentians, Lanaudière, and Montérégie. A random sampling was performed, stratified by region and weighted by the number of pharmacies in each area. Selected pharmacies were sequentially contacted until the target number of 60 was reached. Each participating pharmacy was asked to identify between 10 and 15 consecutive potentially eligible patients. The pharmacists briefly explained the study to the patients and asked them for permission to give their name and phone number to the research team. A research assistant contacted the patients to give them more information about the study procedures and check their eligibility. Patients were selected for the study if they (1) were 18 years of age or older; (2) reported suffering from non-cancer pain for at least 6 months and for a minimum of 2 days per week; (3) rated their average pain in the past 7 days as ≥4 on a 0–10 intensity scale (0 = *no pain*; 10 = *worst possible pain*); (4) had an active analgesic prescription from a primary care physician; (5) spoke and read French or English; and (6) were covered by the RAMQ insurance program for their medication and medical services for a minimum of 292 days (80% of the year) before and after recruitment. The last criterion was necessary to avoid recruiting patients with incomplete health care use information (e.g., due to moving to another province or changing health care insurance program). In addition, patients who died in the year following recruitment were excluded, on the assumption that they were likely to have incurred high health care costs before death^31^ that were unrelated to CNCP. Patients were also excluded if they had attended the participating pharmacy for less than a year, reported migraine or chronic headaches as the sole source of pain, or were unable to provide informed consent due to cognitive deficits.

### Health care use

Patients’ use of health care services and pharmacotherapy was documented for the year preceding and the year following their recruitment into the study, using the MED-ÉCHO and RAMQ databases.

#### Hospitalizations

All-cause hospitalizations were identified in the MED-ÉCHO database, with information collected on date of admission, primary and secondary clinical diagnoses on admission, length of stay, and institution type. For each hospitalization, the primary and secondary medical diagnostic codes specified in the MED-ÉCHO database were reviewed by an experienced pain specialist on the research team (DL) to identify those potentially related to CNCP. All physician consultations (general practitioners and specialists) recorded in the MED-ÉCHO database were documented, along with tests and interventions performed during the hospital stay.

#### Emergency room visits

All emergency room visits and all physician consultations (general practitioners and specialists), tests, and interventions performed during those visits were documented using the MED-ÉCHO database.

#### Ambulatory care

Outpatient physician visits (general practitioners and specialists) and all tests and interventions were documented using the MED-ÉCHO database. Although diagnoses are reported for each medical visit, it is not always possible to reliably identify those related to CNCP.^32^ For this reason, all medical visits had to be considered. However, all tests and intervention codes were reviewed by DL to identify those potentially related to CNCP.

#### Medication use

All analgesics were documented in the RAMQ database along with medications used to treat their common adverse effects. As cited in Table S1, analgesics included acetaminophen, nonsteroidal anti-inflammatory drugs, antidepressants, anticonvulsants, muscle relaxants, opioids, antiviral therapy, disease-modifying antirheumatic drugs, and antirheumatic biologic agents. Antivirals were those recommended for zona-related pain treatment (valacyclovir, famcyclovir, and acyclovir).^33^ Antidepressants and anticonvulsants commonly prescribed for pain treatment^34,35^ were also considered taking into account the dosage used, and we excluded those prescribed by a psychiatrist. Medications prescribed to prevent or control gastrointestinal adverse effects frequently reported with analgesics included laxatives, antacids, gastroprotectives, and antiemetics. For each medication delivered, information was retrieved on date of dispensation, common drug denomination, form, dosage, and quantity.

### Direct health care costs

Direct health care costs were calculated for each patient’s pre- and postrecruitment years.

#### Hospitalizations

Hospitalization costs were the sum of costs related to hospital stay(s), physician visits, and tests/interventions. A per diem cost of CAD 976.24 was attributed to each hospital day (MSSS, 2013 update, unpublished data). This amount was the mean of daily expenses related to hospital-based nursing care, laboratory tests, medications, laundry, food, administration, and maintenance. Costs of physician visits and of tests/interventions performed during hospitalizations corresponded to those reimbursed by the RAMQ in 2013.^36^ Costs of hospitalizations potentially related to CNCP (identified by DL based on primary or secondary clinical diagnosis) were computed.

#### Emergency room visits

The costs of emergency room use, not necessarily related to CNCP, included costs of visits, tests/interventions and physician consultations. A unitary cost of CAD 278.39 (MSSS, 2013 update, unpublished data) was attributed to each emergency room visit. This corresponds to the average cost per emergency room visit and accounts for expenses covered by the provincial health care system.

#### Ambulatory care

Ambulatory care costs were the sum of all costs related to outpatient medical visits, including with general and specialist physicians, that were not CNCP specific, as well as to tests/interventions related to CNCP.

#### Medication use

The costs of prescribed analgesics and medications used to treat common drug side effects corresponded to the amounts reimbursed by the RAMQ, based on the form and dosage of each product. The pharmacist’s fee for each prescription, set by RAMQ, was also included in the costs. Costs were updated to the year 2013.^36^

### Potential predictor variables

Participants’ clinical and psychosocial characteristics were documented using validated questionnaires selected according to the IMMPACT (Initiative on Methods, Measurement, and Pain Assessment in Clinical Trials) group recommendations^37,38^ and with guidance from expert investigators affiliated with the research team. The intention was to gather sufficient data while minimizing respondent burden as much as possible. Information on the recruited patients was collected through a structured telephone interview and a self-administered questionnaire, each with a duration of about 30 min. Trained research assistants carried out all interviews. Sociodemographic and pain characteristics were documented through the telephone interview. Patients’ age, sex, housing status, level of education, working status, and annual family income were recorded. Pain characteristics included duration (in years) and frequency over the past week (continuous or intermittent). Circumstances surrounding the onset and location(s) of pain were also recorded. Pain diagnoses were self-reported in response to the invitation: “I will read you a list of diagnoses that can be at the origin of pain. Please stop me each time I read a diagnosis corresponding to your condition.” The list included ten diagnoses, and patients were given the opportunity to report other diagnoses not included in the list. Diagnoses were then grouped into eight main categories: (1) back pain; (2) neck pain; (3) fibromyalgia; (4) neuropathic pain; (5) visceral pain; (6) inflammatory arthritic pain (e.g., rheumatoid arthritis); (7) degenerative arthritis pain (e.g., osteoarthritis); and (8) tendinitis, bursitis, capsulitis, and epicondylitis. This last variable was used for descriptive purposes only. Both pain diagnosis and location were self-reported. Because self-reported diagnosis was considered less reliable, whereas pain location was viewed as likely to be more accurate, only the latter was entered into the prediction model.

Pain intensity on average and at its worst in the preceding 7 days was assessed with a standard 0–10 numerical rating scale using the descriptors *no pain* and *worst possible pain* as anchors.^38^ Pain-related disability was measured using the mean score on the interference items of the Brief Pain Inventory–10 (BPI-10), which are rated on scale of 0 (*does not interfere at all*) to 10 (*interferes completely*).^39,40^ These items measured the extent to which patients’ pain had interfered in the preceding 7 days with various aspects of their daily living, including general activity, walking ability, mood, normal work, relations with other people, sleep, enjoyment of life, self-care, recreational activities, and social activities.

The self-administered questionnaire was composed of well-validated tools that were assembled into a single instrument. The impact of pain on sleep was assessed using the Chronic Pain Sleep Inventory (CPSI),^41^ which had been translated into French using a back-translation method^42^ as part of an earlier study.^43^ The CPSI contains five items that measure sleep onset, need for sleep medication, awakening because of pain during the night, early morning awakening, and overall quality of sleep. Participants were asked to rate each item on a scale from 0 (*never*) to 10 (*always*). The last item assessed subjective overall sleep quality, rated on a scale from 0 (*very bad*) to 10 (*excellent*). Three of the sleep items (sleep onset, awakening because of pain during the night, and early morning awakening) were summed to obtain the Sleep Quality Index, in which higher scores indicated worse sleep quality.

The Charlson Comorbidity Index^44,45^ was included in the self-administered questionnaire to measure the presence of disorders other than CNCP. This instrument captures the number and severity of disease(s) other than CNCP (e.g., diabetes), with scores ranging from 0 to 10. Patients’ anxiety and depression levels were assessed using the Hospital Anxiety and Depression Scale (HADS), a 14-item Likert scale.^46–48^ A total score, ranging from 0 to 21, was calculated for each of the two subscales; the scores could also be grouped into three categories: *absent* (score ≤7), *uncertain* (scores from 8 to 10), or *probable* (score ≥11).^47^ The Barriers Questionnaire II (BQ-II)^49^ was used to assess patients’ barriers to optimal pain management. The BQ-II contains 27 items rated on a six-point scale anchored with 0 (*do not agree at all*) and 5 (*agree very much*). The items are grouped into four barrier subscales: (1) fear of adverse pathological effects of pain medication (e.g., tolerance); (2) fatalistic beliefs about pain; (3) fear of reporting pain; and (4) concerns about analgesic drug side effects (e.g., addiction). Given that the BQ-II was initially developed for cancer pain patients, some items had to be adapted for CNCP patients. The questionnaire was translated using the back-translation method.^42^

Patients’ attitudes toward pain and its treatment were evaluated using the Survey of Pain Attitudes (SOPA).^50–52^ Patients were asked to rate, on a 0–4 scale (0 = *this is very untrue for me*, 4 = *this is very true for me*), their feelings and attitudes toward pain control, disability, harm, emotions, analgesic medication, solicitude, and medical cures for pain.

Finally, patients’ satisfaction with pain treatment was measured by the mean of the Pain Treatment Satisfaction Scale (PTSS).^53^ The PTSS includes 39 items scored on a five-point Likert scale ranging from 0 to 100; higher scores indicate greater satisfaction. These items are grouped into five domains (satisfaction with current pain medication, medical care, impact of current pain medication, information about pain and its treatment, and side effects of medications).

### Data analyses

For each patient, the total annual direct health care costs in the prerecruitment and postrecruitment year were calculated from the standpoint of the health care system, taking into account the resources used during these periods. Costs were adjusted to 2013 Canadian dollar values based on Statistics Canada consumer price indexes.^36^ They took into account (1) pain-related hospitalizations (identified through primary and secondary diagnoses), which included the corresponding per diem, medical consultations, and tests/interventions; (2) emergency room visits (related and unrelated to CNCP), which included the corresponding unitary costs, medical consultations, and tests/interventions); (3) ambulatory care (related and unrelated to CNCP), which included medical visits and CNCP ambulatory tests/interventions; and (4) pain-related medication. Mean (SD) and median (interquartile range) annual direct health care costs in the postrecruitment year were computed for the entire cohort and were used to define two subcohorts. Both medians and interquartile ranges were reported because of the skewed distribution of health care use and costs. The first cohort, the “heavy health care users,” included patients in the highest quartile of total annual direct health care costs; all other patients were in the second cohort, the “standard health care users.” Likewise, total direct health care costs in the prerecruitment year were classified into two categories: “previous heavy health care users” and “previous standard health care users.”

#### Descriptive analysis

Sociodemographic, clinical, and psychosocial characteristics were described for the entire cohort and for each subcohort using means (SD) for continuous variables and frequency tables for categorical ones.

#### Predictors of heavy health care users

Multivariate logistic regression models were developed to identify the sociodemographic, clinical, and psychological characteristics of patients (independent variables) predicting the risk of being heavy health care users (dependent variable: heavy or standard health care users). The tested predictors included patients’ age, sex, living conditions, working conditions, level of education, annual family income, pain duration (continuous and categorical), pain location and frequency, average and worst pain intensity, pain-related disability, sleep quality, comorbidity levels, anxiety and depression scores (continuous and categorical), patients’ barriers to optimal pain management, patients’ attitudes toward pain and its treatment, and satisfaction with pain treatment. These variables were chosen based on existing literature or on their potential to influence health care use. The Akaike information criterion (AIC)^54,55^ was used to identify the model with the highest predictive power. Because it penalizes the addition of parameters, the AIC is helpful in selecting the model that best fits with a minimum number of parameters (simplicity and parsimony); that is, the model with the lowest AIC value.^56^

All patients with missing data on any of the predictor variables were left out of the regression analysis. The final multivariate logistic model was selected using a forward/backward stepwise approach. First, each potential predictor was tested in a univariate logistic model. The variable leading to the lowest AIC value was entered first into the multivariate model. Thereafter, every other potential predictor was sequentially added to identify the multivariate model with the lowest AIC measure. The same process was replicated until the addition of another variable did not lead to a reduction of the AIC measure. Then, in the backward procedure, each variable was removed from the model, and the variable associated with the largest AIC reduction was eliminated from the multivariate model. This process was applied sequentially until no further AIC reduction was obtained. Finally, the predictive impact of adding into the final predictive model the direct annual health care costs incurred in the prerecruitment year (previous heavy health care users and previous standard health care users) was assessed. The objective was to evaluate how the inclusion of this variable improved the previous predictive model. In a secondary analysis, the same process was conducted after excluding patients reporting rheumatoid arthritis, given that their medication costs are commonly higher than those of patients suffering from other CNCP disorders.

Goodness of fit of the final predictive model was evaluated using the Hosmer-Lemeshow test^57^ and coefficients of determination (pseudo-*R*^2^). The receiver operating characteristic curve was also assessed consecutively at each step. Finally, the adjusted OR and 95% CI of each predictor were calculated (ignoring the selection procedure). The statistical analyses were performed using IBM SPSS Version 19.0 (SPSS Inc, Chicago, IL, USA) and SAS 9.4 (SAS Institute, Cary, NC, USA).

## Results

**Figure 1.**
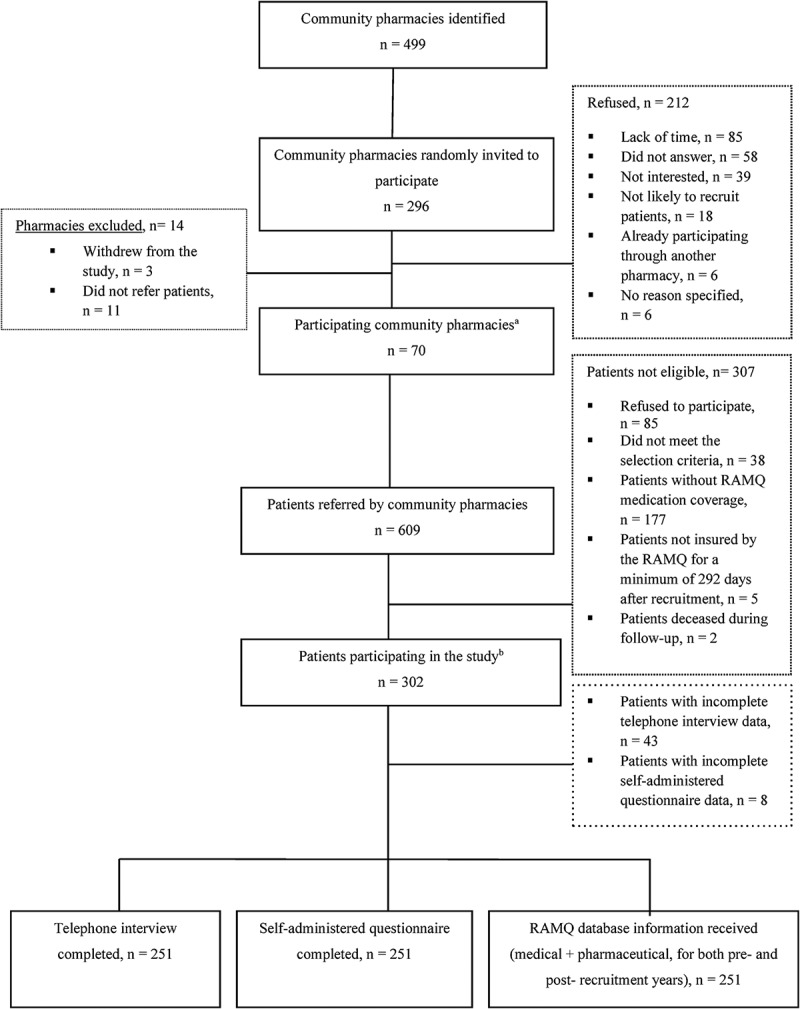
Recruitment of pharmacies and patients. *Notes:* ^a^Pharmacy distribution per region (n of recruited pharmacies/total n per region): *Mauricie et Centre du Québec*, 6/60 (10.0%); Laval, 13/70 (18.6%); Montreal, 18/134 (13.4%); the Laurentians, 14/95 (14.7%); *Lanaudiére*, 8/77 (10.4%); *Montérégie*, 11/77 (14.3%); ^b^Patient distribution per region (n of recruited patients per region/total n of patients): *Mauricie et Centre du Québec*, 44/486 (9.1%); Laval, 67/486 (13.8%); Montreal, 92/486 (18.9%); *Lanaudiére*, 91/486 (18.7%); the Laurentians, 117/486 (24.1%); *Montérégie*, 75/186 (15.4%). Abbreviations: n, number; RAMQ, *Régie de l’Assurance Maladie du Québec*.

As shown in Figure 1, a total of 70 pharmacies referred 609 patients to the study. Of those, 85 (14.0%) declined to participate and 212 (36.5%) were not eligible, including two patients who died during the course of the study (2 and 3 months, respectively, after their recruitment). Fifty-one of the eligible patients (16.9%) were not included the analysis due to incomplete data, mainly for the annual income variable. All remaining participants (*n* = 251) completed both the telephone interview and the self-administered questionnaires. RAMQ and MED-ÉCHO data were available for all of them. Based on total direct health care costs incurred in the postrecruitment year, as described above, 63 heavy and 188 standard health care users were identified.

As shown in Table 1, women made up a majority of the participants (66.5%). Nearly 70% of participants had no more than a high school education. The vast majority were not working, either because they were unable (23.5%) or for other reasons, including staying at home, student, retired, without work, work suspension, and volunteering (63.7%). Nearly 45% of the heavy health care users lived alone, and more than half (50.8%) and had an annual family income below CAD 20,000.

**Table 1.** Sociodemographic characteristics of the participants.

	All patients (n = 251)	Patients stratified by total annual direct health care costs after recruitment	
		Heavy health care users^a^ (*n* = 63)	Standard health care users^b^ (*n* = 188)
Age (years), mean (SD)	61.5 (12.5)	62.5 (10.9)	61.2 (12.5)
Women, *n* (%)	167 (66.5)	43 (68.2)	124 (65.9)
Living, *n* (%)			
Alone	96 (38.3)	28 (44.4)	68 (36.1)
With a partner	99 (39.4)	22 (35.0)	77 (41.0)
Other	56 (22.3)	13 (20.6)	43 (22.9)
Current work status, *n* (%)			
Working	32 (12.7)	7 (11.1)	25 (13.3)
Unable to work	59 (23.5)	16 (25.4)	43 (22.9)
Not working, other reasons^c^	160 (63.7)	40 (63.5)	120 (63.8)
Highest level of education completed, *n* (%)	87 (34.7)	19 (30.2)	68 (36.2)
None or elementary school	87 (34.7)	21 (33.3)	66 (35.1)
High school	36 (14.3)	11 (17.5)	25 (13.3)
College/technical school or CEGEP	41 (16.3)	12 (19.0)	29 (15.4)
University			
Annual family income,^d^ *n* (%)			
<$20 000	110 (43.8)	32 (50.8)	78 (41.5)
$20 000–$50 000	107 (42.6)	24 (38.1)	83 (44.1)
>$50 000	34 (13.6)	7 (11.1)	27 (14.4)

^a^Patients with total annual direct health care costs ≥ CAD 4742 in their postrecruitment year.

^b^Patients with total annual health care costs < CAD 4742 in their postrecruitment year.

^c^Including staying at home, student, retired, without work, work suspension, and volunteering.

^d^Incomes in Canadian dollars. CEGEP = *collège d’enseignement général et professionnel* (schools offering postsecondary technical or pre-university programs in the Quebec system); CAD = Canadian dollars.

Participants’ pain duration varied between 0.5 and 51 years. Table 2 shows that the mean pain duration was 13.1 years (SD = 11.1) in the heavy health care users subcohort and 13.3 years (SD = 12.3) for standard health care users. CNCP was commonly experienced in more than one body area, with the most common site in heavy health care users being the lower body (82.5%), whereas the most frequent site of pain in standard health care users was lumbar, with or without radicular pain (66.5%). About three quarters of the participants had constant pain, with a mean intensity of 6.7 (SD = 1.9) on average in the preceding 7 days. Worst pain intensity and interference scores on the BPI were slightly higher for heavy users than for standard users. Symptoms of probable depression (scores ≥ 11 on the HADS depression subscale) were present in 25.1% of the patients (heavy health care users: 25.4%, standard health care users: 25.0%).

**Table 2.** Clinical and psychosocial characteristics of the participants.

		Patients stratified by total annual direct health care costs after recruitment	
	All patients (n = 251)	Heavy health care users^a^ (*n* = 63)	Standard health care users^b^ (*n* = 188)
Duration of pain (years), mean (SD)	13.2 (11.9)	13.1 (11.1)	13.3 (12.3)
Pain location,^c^ *n* (%)			
Head or neck pain	109 (43.4)	25 (39.7)	84 (44.7)
Upper body pain (shoulders/arms/trapezius/upper back)	136 (54.2)	34 (54.0)	102 (54.3)
Trunk pain (chest/abdomen/middle back)	76 (30.3)	19 (30.2)	57 (30.3)
Lumbar with or without radicular pain	170 (67.7)	45 (71.4)	125 (66.5)
Hip pain	72 (28.7)	22 (34.9)	50 (26.6)
Lower body pain (buttocks/legs)	172 (68.5)	52 (82.5)	120 (63.8)
Diffuse pain (≥5 pain sites of pain)	36 (14.3)	11 (17.5)	25 (13.3)
Self-reported pain diagnoses,^d^ *n* (%)			
Chronic back and/or neck pain	187 (74.5)	49 (77.8)	137 (72.9)
Fibromyalgia	61 (24.3)	18 (28.6)	43 (22.9)
Osteoarthritis, arthrosis and others	173 (68.9)	52 (82.5)	121 (64.4)
Inflammatory arthritis, rheumatoid and others	31 (12.4)	14 (22.2)	17 (9.0)
Tendinitis, bursitis, capsulitis, and epicondylitis	43 (17.1)	9 (14.3)	34 (18.1)
Others	56 (22.3)	17 (22.4)	39 (20.7)
Frequency of pain in the past 7 days, *n* (%)			
Continuous	191 (76.1)	46 (73.0)	145 (77.1)
Intermittent	60 (23.9)	17 (27.0)	43 (22.9)
Average pain intensity in the past 7 days^e^ (NRS), mean (SD)	6.7 (1.9)	7.1 (1.8)	6.6 (1.9)
Worst pain in the past 7 days,^e^ (NRS), mean (SD)	8.2 (1.9)	8.6 (1.4)	8.0 (2.0)
Pain-related disability (Interference score on the BPI),^f^ mean (SD)	5.4 (2.3)	6.0 (2.3)	5.2 (2.3)
Sleep Quality Index on the CPSI,^g^ mean (SD)	5.1 (3.1)	5.6 (3.4)	5.0 (3.0)
Charlson Comorbidity Index,^h^ mean (SD)	2.9 (1.9)	3.3 (1.8)	2.8 (1.9)
Depression score on the HADS, mean (SD)^i^	7.5 (4.1)	6.9 (4.5)	7.7 (4.0)
Anxiety score on the HADS, mean (SD)^j^	9.4 (4.5)	9.2 (4.6)	9.5 (4.5)
Barriers to optimal pain management (scores on the BQ-II subscales),^k^ mean (SD)			
Fear of adverse physiological effects of pain medication	2.5 (0.8)	2.5 (0.9)	2.5 (0.8)
Fatalistic beliefs about pain	3.0 (1.2)	3.2 (1.4)	2.9 (1.1)
Fear of reporting pain	2.3 (1.2)	2.3 (1.2)	2.3 (1.1)
Concerns about analgesic drug side effects	3.1 (1.3)	3.2 (1.4)	3.1 (1.3)
Attitudes toward pain and its treatment (scores on the SOPA subscales), mean (SD)^l^			
Pain control	1.7 (1.0)	1.8 (1.1)	1.7 (1.0)
Disability	2.5 (1.3)	2.4 (1.3)	2.5 (1.3)
Harm	2.6 (1.0)	2.5 (1.0)	2.6 (0.9)
Emotions	2.1 (1.2)	2.1 (1.2)	2.1 (1.2)
Medication	3.6 (0.7)	3.6 (0.6)	3.6 (0.7)
Solicitude	1.7 (1.2)	1.5 (1.2)	1.8 (1.3)
Medical pain cure	1.4 (1.1)	1.7 (1.2)	1.4 (1.1)
Pain treatment satisfaction (scores on the PTSS subscales),^m^ mean (SD)			
Satisfaction with pain medication	63.5 (17.2)	62.9 (16.6)	63.7 (17.4)
Satisfaction with medical care	67.1 (17.6)	67.2 (18.3)	67.0 (17.6)
Impact of current pain medication	58.1 (23.1)	60.7 (24.2)	57.2 (22.7)
Satisfaction with side effects of the medication	73.6 (17.8)	74.1 (16.9)	73.4 (18.2)
Satisfaction with information about pain and its treatment	49.6 (34.6)	51.1 (34.6)	49.1 (34.7)

^a^Patients with total annual direct health care costs ≥ CAD 4742 in their postrecruitment year.

^b^Patients with total annual direct health care costs < CAD 4742 in their postrecruitment year.

^c^Individuals could report more than one pain site.

^d^Diagnosis self-reported by individuals; they could report more than one.

^e^0–10, 10 = worst pain.

^f^0–10, 10 = worst disability.

^g^0–10, 10 = worst sleep.

^h^ 0–10, with higher scores indicating greater comorbidity.

^I^0–21, 21 = worst depression.

^j^0–21, 21 = worst anxiety.

^k^0–5, with higher scores indicating greater barriers to pain management.

^l^0–4, with higher scores indicating a better attitude to pain.

^m^0–100, with higher scores indicating greater treatment satisfaction. NRS = Numeric Rating Scale; BPI = Brief Pain Inventory; CPSI = Chronic Pain Sleep Inventory; HADS = Hospital Anxiety and Depression Scale; BQ-II = Barrier Questionnaire II; SOPA = Survey of Pain Attitudes; PTSS = Pain Treatment Satisfaction Scale; CAD = Canadian dollars.

The annual rates of hospitalization for CNCP were higher among the heavy health care users in the postrecruitment year than for the standard users (31/63: 49% versus 23/188: 12%). As shown in Table 3, heavy health care users had a larger interquartile range for pain-related hospitalisation days compared to the standard health care users (4 versus 0 days). They also had more emergency room and ambulatory care visits and were prescribed more analgesics and medications to treat side effects.

**Table 3.** Annual health care resources used per patient during the postrecuitment year.

	Heavy health care users^a^ (n = 63)	Standard health care users^b^ (n = 188)
	Median (IR)/% null use^c^ (IR)	Median (IR)/% null use^c^ (IR)
Hospitalizations		
Number of pain-related hospitalization days^d^	50.8% (4)	87.8% (0)
Number of physician visits during hospitalizations		
General practitioners	63.5% (3)	90.4% (0)
Specialists	54.0% (2)	89.4% (0)
Emergency room visits		
Number of emergency room visits	1 (3)	75.5% (0)
Number of physician visits at emergency		
General practitioners	1 (4)	79.8% (0)
Specialists	65.1% (1)	93.6% (0)
Ambulatory care		
Number of physician visits		
General practitioners	4 (7)	3 (5)
Specialists	3 (6)	1 (3)
Number of outpatient pain-related tests/interventions	1 (4)	62.7% (2)
Number of prescribed analgesics and medications to treat their side effects	6 (3)	4 (3)

^a^Patients with total annual direct health care costs ≥ CAD 4742 in their postrecruitment year.

^b^Patients with total annual direct health care costs < CAD 4742 in their postrecruitment year.

^c^When median use was equal to zero, percentage of patients with zero use and IR were both reported.

^d^Hospitalizations with a primary or secondary clinical diagnosis related to chronic non-cancer pain. IR = interquartile range; CAD = Canadian dollars.

Table 4 presents the total annual costs by expenditure category for the two subcohorts in the postrecruitment year. The total direct health care cost was estimated at CAD 600 012 for the 63 heavy health care users and CAD 300 800 for the 188 standard health care users. In both groups, more than half of total costs were attributable to prescribed pain-related medications (heavy health care users: 62.2%; standard health care users: 52.7%). Hospitalization costs accounted for 24.6% and 22.8% of the total costs for heavy and standard health care users, respectively.

**Table 4.** Total annual costs^a^ by health care expenditure category for all patients.^a^

	Heavy health care users^b^ (n = 63)		Standard health care users^c^ (n = 188)	
Health care expenditure category	Total costs	% of overall total direct cost	Total costs	% of overall total direct cost
Hospitalizations				
Pain-related hospitalization costs excluding physician visits^d,e^	140,490	23.4	63,920	21.3
General practitioner visits	2,709	0.5	1,128	0.4
Specialist visits	4,410	0.7	3,196	1.1
Total costs	147,609	24.6	68,244	22.8
Emergency room visits				
Emergency room costs^e^	40,824	6.8	22,936	7.6
General practitioner visits	3,591	0.6	2,256	0.8
Specialist visits	4,662	0.8	1,504	0.5
Total costs	49,077	8.2	26,696	8.9
Ambulatory care				
General practitioner visits	12,600	2.0	22,748	7.6
Specialist visits	14,805	2.5	19,928	6.6
Pain-related tests/interventions	2,772	0.5	7,144	2.4
Total costs	30,177	5.0	49,820	15.6
Prescribed analgesics and medication to treat their side effects	373,149	62.2	156,040	52.7
Total health care costs	600,012	100	300,800	100

^a^Prices in CAD, adjusted for the year 2013.

^b^Patients with total annual direct health care costs ≥ CAD 4742 in their postrecruitment year.

^c^Patients with total annual direct health care costs < CAD 4742 in their postrecruitment year.

^d^Hospitalizations with a primary or secondary clinical diagnosis related to chronic non-cancer pain.

^e^Costs of tests/interventions included. CAD = Canadian dollars.

In the heavy health care users, total annual direct costs per patient ranged from CAD 4742 to CAD 58 832, with a median of CAD 7627 (Table 5). Corresponding costs for the standard health care users ranged from CAD 81 to CAD 4657, with a median of CAD 1554. The difference was mainly in pain-related hospitalization costs, which were $2343 for heavy users and $363 for standard users, and prescribed medication costs of $5923 and $830, respectively.

**Table 5.** Annual direct health care costs^a^ per patient during the postrecruitment year.

	Heavy health care users^b^ (n = 63)	Standard health care users^c^ (n = 188)
	Median (IR)/% null cost^d^ (IR)	Median (IR)/% null cost^d^ (IR)
Hospitalizations		
Pain-related hospitalization costs excluding physician visits^e,f^	50.8% (1,990)	87.8% (0)
General practitioner visits	63.5% (143)	90.4% (0)
Specialist visits	54.0% (190)	89.4% (0)
Total costs	50.8% (2,150)	87.4% (0)
Emergency room visits		
Emergency room costs^e^	278 (896)	75.5% (0)
General practitioner visits	13 (92)	79.8% (0)
Specialist visits	65.1% (89)	93.6% (0)
Total costs	323 (1,094)	75.5% (0)
Ambulatory care		
General practitioners visits	157 (231)	93 (185)
Specialist visits	139 (309)	53 (151)
Pain-related tests/interventions	22 (78)	62.7% (43)
Total costs	439 (489)	177 (300)
Prescribed analgesics and medication to treat their side effects	4,941 (5,841)	902 (1,276)
Total health care costs	7,627 (6,175)	1,554 (1,761)

^a^Prices in CAD, adjusted for the year 2013.

^b^Patients with total annual direct health care costs ≥ CAD 4742 in their postrecruitment year.

^c^Patients with total annual direct health care costs < CAD 4742 in their postrecruitment year.

^d^When median cost was equal to zero, percentage of patients with zero cost and IR were both reported.

^e^Hospitalizations with a primary or secondary clinical diagnosis related to chronic non-cancer pain.

^f^Costs of tests/interventions included. IR = interquartile range; CAD = Canadian dollars.

Table 6 shows the predictive ability of the logistic regression models developed for identifying, among the sociodemographic, clinical, and/or psychological variables listed in Tables 1 and 2 (except for self-reported diagnosis), those that predicted heavy health care use. At the end of the forward procedure, six variables were retained in the regression model and provided the best prediction, with an AIC of 252.1, a discriminatory power of 70.7%, and an adjusted pseudo-*R*^2^ of 17.1% (model 6, Table 6). These variables were (1) sex, (2) lower body pain, (3) pain-related disability, (4) comorbidity levels, (5) depression levels, and (6) patient’s attitude toward medical pain cure. In the backward selection process, AIC was not further reduced by the removal of any of these variables.

**Table 6.** Predictive ability of the logistic models using a forward/backward stepwise approach for selecting the variables.^a^

Model no.	Variables	Maximum likelihood function (−2logL)	AIC	C (%)	*R*^2^ (%)
Stepwise: Forward					
1	Pain in lower body	274.6	278.6	59.4	4.8
2	Pain-related disability, pain in lower body	269.4	275.4	65.1	7.7
3	Depression level,^b^ pain-related disability,^b^ pain in lower body	263.1	273.1	67.8	11.2
4	Charlson comorbidity index,^b^ depression level,^b^ pain-related disability,^b^ pain in lower body	257.9	267.9	69.0	14.0
5	Sex, Charlson Comorbidity Index,^b^ depression level,^b^ pain-related disability,^b^ pain in lower body	254.6	266.6	70.9	15.7
6	Attitudes toward pain medical cure, sex, Charlson Comorbidity Index,^b^ depression level,^b^ pain-related disability,^b^ pain in lower body	252.1	266.1	70.7	17.1

^a^Attitudes toward medical pain cure measured by the Survey of Pain Attitudes; depression level measured on the Hospital Anxiety and Depression Scale.

^b^Only scale variable retained. logL = log-Likelihood function; AIC = Akaike information criterion; C = area under the receiver operating characteristic curve; *R*^2^ = pseudo-*R*^2^ of Nagelkerke.

Health care user type in the prerecruitment year was entered last into the predictive model. Of the 63 heavy health care users in the postrecruitment year, 65% (41/63) had also been heavy users in the prerecruitment year, whereas only 9% (17/188) of the postrecruitment standard health care users had been heavy users in the prerecruitment year. When this variable was added into the predictive model, AIC was further decreased to 198.1 and discriminatory power increased to 85.9%, whereas the pseudo-*R*^2^ reached 46.0%.

As shown in Table 7, patients who had been heavy health care users prior to recruitment into the study were more likely remain so after recruitment (OR = 17.7, 95% CI, 7.90–39.48). Lower body pain (OR = 3.03, 95% CI, 1.20–7.65) and greater pain-related disability (OR = 1.24, 95% CI, 1.03–1.48) were also significant independent predictors of higher risk of being a heavy health care user. All remaining variables (sex, comorbidity, depression levels, attitude toward medical cure) were useful in predicting heavy health care use, but their respective ORs did not reach statistical significance after entering the variable “type of health care user prior to recruitment” into the model.

**Table 7.** Predictors of heavy health care use with the indicator of prerecruitment heavy/standard health care users: final multivariate regression model.

Predictors	Odds ratio	95% CI
Sociodemographic predictors:		
Sex		
Male	—	—
Female	1.23	0.55–2.77
Clinical and psychosocial predictors:		
Pain located in lower body (buttocks/legs)		
No	—	—
Yes	3.03	1.20–7.65
Pain-related disability (Interference score on the BPI) (0–10)	1.24	1.03–1.48
Charlson Comorbidity Index (0–10)	1.17	0.97–1.42
Depression score on the HADS (0–21)	0.92	0.83–1.02
Attitudes toward pain and its treatment		
(scores on the SOPA subscales): medical pain cure (0–4)	1.21	0.86–1.70
Type of health care users prior to recruitment		
Previous standard health care users	—	—
Previous heavy health care users	17.67	7.90–39.48

CI = confidence interval; BPI = Brief Pain Inventory; HADS = Hospital Anxiety and Depression Scale; SOPA = Survey of Pain Attitudes; — = reference category

Finally, when patients suffering from rheumatoid or other types of inflammatory arthritis were excluded from the above analyses, the prerecruitment health care user type (heavy versus standard) remained the best predictor of heavy health care use in the postrecruitment year (OR = 2.96, 95% CI, 1.46–6.00). The corresponding OR remained statistically significant even though it was considerably decreased. Lower body pain and greater pain-related disability also continued to be significantly associated with heavy health care use in the postrecruitment year (OR = 2.58, 95% CI, 1.13–5.88; OR = 1.24, 95% CI, 1.03–1.50, respectively). Other variables entered into the final model for which the OR did not reach statistical significance were sex, depression, pain duration, pain intensity, and comorbidity index (results not shown).

## Discussion

To our knowledge, this study is the first to use a longitudinal design to identify the sociodemographic, psychosocial, and clinical characteristics predicting the risk of being heavy health care users among CNCP patients recruited in primary care. Patients with pain in the lower body and presenting greater pain-related disability were found to be significantly more at risk of making greater use of health care resources and generating higher direct costs for the health care system. Use of health care resources in the prerecruitment year was also an important predictor of use in the postrecruitment year, even after excluding patients suffering from rheumatoid or other types of inflammatory arthritis.

This study confirms the substantial economic burden of CNCP on our health care system. Direct costs were mainly associated with pain-related hospitalizations and prescribed medications—that is, analgesics and drugs to treat analgesic side effects—regardless of whether patients were heavy or standard health care users. It is important to recognize that the high direct health care costs of CNCP found in the present study represent only the tip of the iceberg. To these must be added costs associated with work productivity losses (absenteeism, presenteeism) common in CNCP patients^11^ and costs incurred for complementary/alternative pain treatment.^11–13^ The present study was not aimed at assessing predictors of substantial health care use, including complementary health care services (which are not covered by Quebec’s public health care system), but it would be interesting to do so in a future study. In particular, Lalonde et al. found that complementary health care services accounted for almost 50% of direct health care costs in CNCP primary care patients.^11^ This was also observed in tertiary care patients by Guerrière et al., who found that 95% of total expenditures related to CNCP were privately financed.^12^

CNCP often has adverse impacts on various aspects of patients’ daily functioning.^11,58^ Our study suggested that lower body pain and greater pain-related disability were independent predictors of heavy health care use over a 1-year period. Comparable results were reported for chronic back pain by Engel et al.,^59^ who found, after adjusting for sex, education, pain duration, depression, and pain etiology, that when pain disability worsened from level I (low) to level IV (high), patients’ risk of incurring high health care costs increased approximately fivefold. In a recent study, Lalonde et al.^11^ observed a significant positive association between severity of pain-related disability in CNCP patients and total direct health care costs, after adjusting for age, sex, pain duration, and comorbidity. Hogan et al. also found similar results but using an incremental approach for calculating direct costs associated with chronic pain management.^20^ In light of the above results, it appears that deterioration in patients’ functioning due to CNCP can translate into more health care use and consequently higher direct costs. Disability in chronic diseases other than CNCP was analyzed by Manton et al.,^60^ who also showed that it was an important driver of health care costs.

Another parameter retained in the final prediction model was sex. However, the associated OR failed to reach statistical significance, most likely because its variance was largely accounted for by patients’ health care use in the prerecruitment year. In an earlier study involving patients referred to tertiary care pain clinics, Weir et al.^61^ observed that women incurred on average higher total direct pain-related costs than men. However, two recent studies^62,63^ failed to find such a sex difference in similar patient populations. Additional studies are clearly needed to further investigate the association between sex and CNCP health care costs in primary care patients.

Another aspect to consider in assessing CNCP costs is the presence and severity of comorbidity. Many patients with CNCP suffer from other chronic but nonpainful diseases such as cardiovascular diseases and diabetes,^64^ multiple sclerosis,^65^ insomnia,^66^ and mental disorders, including anxiety and depression disorders.^6,67–69^ In our study, patient’s comorbidity level was found to be a predictor of health care use. Our results are consistent with those reported in a Swiss study that included patients suffering from osteoarthritis, back pain, and fibromyalgia, whose comorbidity level was measured with the Self-administered Comorbidity Questionnaire.^70^ That study found that patients’ comorbidity levels increased direct health care costs. Likewise, in a sample of patients suffering from osteoarthritis followed in primary care, a higher comorbidity score, measured by the Charlson Comorbidity Index, was found to increase the risk of incurring higher direct health care costs.^26^ Thus, comorbidity appears to be an important driver of health care costs. The nonsignificance of its associated OR in our final predictive model is likely due to the fact that prerecruitment health care use would represent a good proxy of patients’ comorbidities.

Depression disorders are common in patients suffering from CNCP,^67,71–73^ and several studies^12,21,22^ have shown that CNCP patients suffering from depression tend to use more health care services and incur higher costs than those who are not depressed. Our study failed to replicate these results and may even suggest the opposite. We have no explanation for this finding, except for the possibility that depression levels and pain-related health care are not linearly related. The hypothesis that CNCP patients with severe depression and low energy levels tend to isolate themselves to the point of seeking out less and less medical treatment for their pain warrants further investigation.

The present study suggests that patients who expect a medical cure for their CNCP tend to incur higher health care costs. In another study, Merkesdal and Mau^28^ also found in a sample of patients suffering from chronic low back pain that those whose expectations regarding an outpatient rehabilitation program were low used more health care resources. These results are interesting in that patients’ attitudes toward chronic pain and its treatment is a factor that can be modified by simple education programs or cognitive–behavioral therapy.^74,75^ Although there is an abundant literature on factors associated with patients’ tendency to catastrophize in the face of pain,^76–78^ further research is needed to understand the determinants of expectations regarding pain treatment.

We believe that our study comprehensively describes the direct health care costs for primary care patients with CNCP. We also assessed a variety of sociodemographic, clinical, and psychological factors that may put patients at risk of being heavy health care users, with a rigorous statistical procedure^79,80^ designed to identify the most parsimonious model of predictors among a set of models.

Like any other study, this one has limitations. First, our results cannot be generalized to populations of CNCP patients other than those suffering moderate or severe pain who have an analgesic prescription from a primary care. Second, some of our results are based on patient self-reports that may have been influenced by social desirability factors and/or memory biases. However, the research assistants who interviewed the patients by telephone were carefully trained and used a structured interview protocol. Other self-reported measures were collected with validated questionnaires that are widely used in the field of pain research. Another limitation concerns the methods used to derive health care costs. Although records in the MED-ÉCHO administrative database regarding hospitalizations, emergency room visits, ambulatory care visits, and tests/interventions are objective and not subject to recall biases, it was not always easy and sometimes impossible to determine whether these data were specifically related to CNCP. The same was true for prescribed antidepressant and anticonvulsant medications commonly used in CNCP treatment (RAMQ data). We chose to include in our final analysis pain-related data only on hospitalizations, outpatient tests/interventions, and medications. For other categories of health care use, it was not possible to distinguish whether they were pain related or not. This may have resulted in overestimation of CNCP direct health care costs. Whether the magnitude of the overestimation is the same in both patient cohorts (heavy and standard health care users) is unknown. A last limitation of our study has to do with the time frame. It may be that examining health care costs over a period greater than 2 year before and after patients’ recruitment into the study would have yielded a different pattern of results in terms of predictors of heavy health care use.

## Conclusion

The present study revealed that that CNCP patients suffering from lower body pain and showing greater pain-related disability were more likely to be heavy health care users even after adjusting for their previous-year direct health care costs. Improving pain management in this patient clientele could help to decrease their health care use and the associated costs.

## Disclosures

Between 2009 and 2012, Manon Choinière was member of the Scientific Committee of the Pfizer Neuropathic Pain Award and received honoraria from Pfizer Canada Inc. for reviewing grant applications and assisting to the meetings of the committee.

All authors of the present article certify that they have no conflict of interest with any financial organization regarding the material presented and discussed in this article.

## Supplementary Material

1326088_Supplemental_Material.docx
