# Supplementary material for: Identifying heavy health care users among primary care patients with chronic non-cancer pain
Source: Can J Pain. 2017 Jul 6;1(1):22–36. doi: 10.1080/24740527.2017.1326088 (PMC8730606; doi:10.1080/24740527.2017.1326088)
Supplement: 1326088_Supplemental_Material.docx [file UCJP_A_1326088_SM6207.docx]

| Supplementary material | |
| --- | --- |
| Table S1 - Documented analgesics | |
| Therapeutic class International | **common denomination** |
| Anticonvulsants |  |
| Carboxylic acid derivatives | Valproic acid |
| Gamma-aminobutyric acid analogs | Gabapentin, Pregabalin |
| Iminostilbene | Carbamazepine, Oxcarbazepine |
| Other anticonvulsants | Divalproex, Lamotrigine, Topiramate |
| Antidepressants |  |
| Selective serotonin reuptake inhibitors | Citalopram, Escitalopram, Fluoxetine, Fluvoxamine, Paroxetine, Sertraline, Trazodone |
| Serotonin and noradrenaline reuptake inhibitors | Duloxetine, Venlafaxine |
| Tricyclic and tetracyclic antidepressants | Amitriptyline, Clomipramine, Desipramine, Doxepin, Imipramine, Maprotiline, Mirtazapine, Nortriptyline, Trimipramine |
| Other antidepressants | Bupropion |
| Antiretroviral | Acyclovir, Famciclovir, Valacyclovir |
| Disease-modifying antirheumatic drugs | Auranofin, Aurothioglucose, Aurothiomalate, Azathioprine, Chloroquine, Cyclosporine, D-penicillamine, Hydroxychloroquine, Leflunomide, Methotrexate, Sulfasalazine |
|  |  |
| Muscle relaxants |  |
| Benzodiazepine | Diazepam, Tetrazepam |
| Carbamic acid derivatives | Methocarbamol |
| Centrally acting myorelaxants | Cyclobenzaprine, Tizanidine |
| Gamma-aminobutyric acid derivatives | Baclofen |
| Other muscle relaxants | Thiocolchicoside |
| Nonsteroidal antiinflammatory |  |
| Acetic acids | Diclofenac, Etodolac, Indomethacin, Ketorolac, Nabumetone |
| Acetylsalicylic acid |  |
| Coxibs | Celecoxib Fenamates Meclofenamate, Mefenamic acid |
| Oxicams | Meloxicam, Piroxicam |
| Propionic acids | Fenoprofen, Flurbiprofen, Ibuprofen, Ketoprofen, Naproxen, Oxaprozin |
| Opioids |  |
| Agonist–antagonist derivatives | Buprenorphine, Butorphanol, Nalbuphine, Pentazocine |
| Antagonists | Naloxone |
| Benzomorphan derivatives | Pentazocine |
| Centrally acting analgesics | Tramadol |
| Meperidine-like agonists | Fentanyl, Meperidine |
| Methadone-like agonists | Methadone, Propoxyphene |
| Morphine-like agonists | Codeine, Hydrocodone, Hydromorphone, Levorphanol, Morphine, Oxycodone, Oxymorphone |
| Natural cannabinoids | Tetrahydrocannabinol, Cannabidiol |
| Synthetic cannabinoids | Dronabinol, Nabilone |
|  |  |
